# Mesenchymal Stem Cell Plasticity: What Role Do Culture Conditions and Substrates Play in Shaping Biomechanical Signatures?

**DOI:** 10.3390/bioengineering11121282

**Published:** 2024-12-17

**Authors:** Marina Danalache, Lena Karin Gaa, Charline Burgun, Felix Umrath, Andreas Naros, Dorothea Alexander

**Affiliations:** 1 Department of Orthopedic Surgery, University Hospital Tübingen, 72072 Tübingen, Germany; marina.danalache@med.uni-tuebingen.de; 2 Department of Oral and Maxillofacial Surgery, University Hospital Tübingen, 72076 Tübingen, Germany; lena@dresgaa.de (L.K.G.); charline.burgun@gmx.de (C.B.); felix.umrath@med.uni-tuebingen.de (F.U.); andreas.naros@med.uni-tuebingen.de (A.N.)

**Keywords:** mesenchymal stem cells, culture condition, mechanobiology, laminin and gelatin coatings, calcium phosphate precipitates, atomic force microscopy, cellular stiffness, Young’s modulus

## Abstract

Cell functionality, driven by remarkable plasticity, is strongly influenced by mechanical forces that regulate mesenchymal stem cell (MSC) fate. This study explores the biomechanical properties of jaw periosteal cells (JPCs) and induced mesenchymal stem cells (iMSCs) under different culture conditions. We cultured both JPCs and iMSCs (n = 3) under normoxic and hypoxic environments, with and without osteogenic differentiation, and on laminin- or gelatin-coated substrates. Using atomic force microscopy, we measured cellular elasticity and Young’s modulus of calcium phosphate precipitates (CaPPs) formed under osteogenic conditions. Correlation analyses between cellular stiffness, quantity of CaPP deposition, and stiffness of formed CaPPs were evaluated. The results showed that iMSCs, despite their softer cellular consistency, tended to form CaPPs of higher elastic moduli than osteogenically differentiated JPCs. Particularly under normoxic conditions, JPCs formed stronger CaPPs with lower cellular stiffness profiles. Conversely, iMSCs cultivated under hypoxic conditions on laminin-coated surfaces produced stronger CaPPs while maintaining lower cellular stiffness. We conclude that JPCs and iMSCs display distinct biomechanical responses to culture conditions. While JPCs increase cellular stiffness during osteogenic differentiation, in particular under hypoxic conditions, iMSCs exhibit a decrease in stiffness, indicating a higher resistance to lower oxygen levels. In both cell types, a lower cellular stiffness profile correlates with enhanced mineralization, indicating that this biomechanical fingerprint serves as a critical marker for osteogenic differentiation.

## 1. Introduction

Mesenchymal stem cells (MSCs) undergo morphological changes during differentiation, with a particular emphasis on Young’s modulus, which quantifies a material’s/cell’s stiffness. An increased Young’s modulus indicates enhanced stiffness and a reduced capacity for deformation. Previous investigations have explored the modifications in MSC stiffness that occur post-differentiation or in response to fluctuations in temperature [1,2,3]. The mechanical properties of MSCs are dictated by their cytoskeletal networks [4], which are essential for the generation, transmission, and cellular responsiveness to mechanical signals. The disruption of the actin cytoskeleton with cytochalasins leads to a significant decrease in cellular stiffness and an increase in viscosity [3]. In contrast, disrupting microtubules with nocodazole does not significantly affect stiffness, suggesting that the stiffness of MSCs is primarily dependent on the actin cytoskeleton [3]. Two non-mechanical factors that influence the structure of the actin cytoskeleton in MSCs are the inhibition of the perinuclear actin cap and cell population doubling [5]. Notably, the mean ratio of the MSCs of passage 6 with a collapsed actin cytoskeleton treated with the actin-stabilizing drug jasplakinolide was found to be lower than in those of passage 2 [6]. The authors of the study explained this observation with the decreased actin turnover and increased levels of the actin cross-linking protein transgelin in old MSCs. Therefore, it is recommended to use MSCs at low passages to ensure cytoskeletal integrity [7]. Sheyn and colleagues claim that the frequency of MSCs in the tissue of older patients hardly changes, but the ability for self-renewal and functionality in tissue regeneration does decline negatively [8]. MSCs can be obtained from many different tissues in the body; however, the yield is usually low [9]. Consequently, isolated MSCs require substantial in vitro expansion, which frequently results in the emergence of replication-induced senescence [10].

Jaw periosteal cells (JPCs) possess, like all other periosteal cells in the human body, bone regenerative capacity. Since JPCs are exposed to high mechanical loads triggered by the mastication process, these cells are ideally suited for regenerative purposes in oral and maxillofacial surgery. To create a more effective, ideally rejuvenated alternative to jaw periosteal cells previously employed in our tissue engineering constructs, while also mitigating donor variability, we reprogrammed these cells into induced pluripotent stem cells (iPSCs) [11]. Through a stepwise differentiation process, we efficiently derived mesenchymal stem cells (iMSCs) from iPSCs [12]. Although these rejuvenated iMSCs exhibited distinct functional differences compared to the original JPCs, they preserved their osteogenic differentiation potential. Our previous research established that culture conditions significantly influence the mineralization potential of iMSCs and JPCs [12,13]. Consequently, we hypothesize that, in addition to the culture medium, factors such as dish format, laminin and gelatin coatings, and the modulation of normoxic versus hypoxic environments play pivotal roles in determining the differentiation potential and functionality of JPCs. Since normoxic culture conditions are quite artificial, and in vivo measurements of local oxygen tension (pO_2_) within bone tissues detected values varying between 1 and 7%, with the highest levels detected in the periosteum [14], JPCs are supposed to be quite susceptible to hypoxic conditions. In this context, it was of interest for us to analyze whether this feature remains memorized after cell reprogramming.

Cellular stiffness reflects the biomechanical rigidity of a cell and is quantitatively assessed using Young’s modulus, which measures the elastic resistance of the cell to deformation under applied stress. Higher Young’s modulus values indicate stiffer cells, while lower values correspond to more compliant cells. This parameter not only reflects the cell’s structural integrity (regulated by the cytoskeletal architecture) but also serves as a window into its physiological state and mechanotransduction processes [15]. Cellular stiffness plays a pivotal role in osteogenic differentiation, as increased topography-induced stiffness often enhances mechanotransduction pathways that promote the expression of osteogenic markers and matrix mineralization [16,17]. Therefore, we hypothesize that changes in environmental and cultivation factors directly influence the cellular mechanical fingerprint, shaping its biomechanical properties.

In this study, we systematically compared iMSCs with their parental JPCs, cultured under varying conditions, to assess the impact of these culture environments on their biochemical and biomechanical fingerprints and implicitly on their functionality. Additionally, we investigated the relationship between cellular elasticity and the inorganic precipitates formed to elucidate the optimal culture conditions for enhancing cellular performance for future clinical applications.

## 2. Materials and Methods

**Figure 1:**
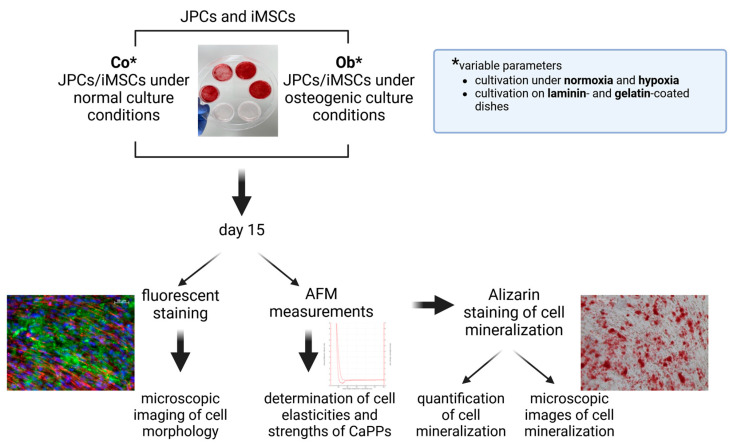
Experimental design flow chart. Jaw periosteal cells (JPCs) and induced mesenchymal stem cells (iMSCs) were cultured under varying conditions (normoxia/hypoxia, laminin- and gelatin-coated plates) in both control (Co) and osteogenic (Ob) environments. After 15 days of cultivation, we performed in parallel dishes fluorescent staining and atomic force microscopy (AFM) measurements, as well as histological Alizarin staining in dishes used for AFM, to assess cell mineralization. * Untreated (Co) and osteogenically (Ob) induced JPCs/iMSCs were cultured und normoxia/hypoxia on laminin- or gelatin-coated dishes.

The experimental workflow is illustrated in Figure 1. For our experimental approach, we used jaw periosteal cells (JPCs) and induced mesenchymal stem cells (iMSCs), which were generated from reprogrammed JPCs. Either untreated/control (Co) or osteogenically differentiated (Ob) cells were cultivated under different culture conditions: under normoxia versus hypoxia and on laminin or gelatin-coated dishes (as listed in the blue text field of the figure). After day 15 of in vitro cultivation, fluorescent staining for the imaging of cell morphology, atomic force microscopic (AFM) measurements for determination of cell and precipitate elasticities, and Alizarin staining for detection and quantification of cell mineralization were carried out.

### 2.1. Cell Culture

JPCs derived from three donors were included in this study in accordance with the local ethical committee (approval number 618/2017BO2) and after obtaining written informed consent. Jaw periosteal tissue was extracted during routine surgery and JPCs were isolated and expanded as previously reported [18,19]. JPCs and iMSCs were grown in hPL5-medium (DMEM/F12 (Gibco, Waltham, MA, USA) containing 5% human platelet lysate (hPL, ZKT Tübingen GmbH, Tübingen, Germany), 100 U/mL penicillin–streptomycin (Pen-Strep, Lonza, Basel, Switzerland), and 2.5 µg/mL amphotericin B (Biochrom, Berlin, Germany)). For the herein-described experiments, cell culture plates were coated with either 0.1% porcine gelatin (Sigma-Aldrich, St. Louis, MO, USA) in ddH2O or with 0.1 mg/mL recombinant human laminin-521 (Thermo Fisher Scientific Inc., Waltham, MA, USA) in PBS with Ca and Mg according to the manufacturer’s instructions.

The limited sample size (n = 3 donors) in this study reflects standard practices for exploratory research involving human biological material, where availability and variability often pose practical constraints. While this inherently limits the generalizability of the findings, the observed trends align closely with previously published data [20,21,22], suggesting consistency across independent studies. Future investigations with larger cohorts will be essential to validate these findings and further enhance their robustness and applicability. However, we note high numbers of AFM data, as indicated in the figure legends.

### 2.2. Generation of iMSCs

JPCs were reprogrammed to iPSCs as previously published [11]. For iMSC differentiation, iPSCs were grown in 6-well plates and detached using a cell scraper on day 0 of iMSC differentiation. Cell aggregates were transferred into VTN-coated T75 flasks containing 10 mL of hPL5 medium with 10 µM Y27632. Cells were then labeled as iMSCs passage 0 (P0). On day 1, medium was changed to hPL5 medium containing 10 μM SB431542 (Selleck Chemicals LLC, Houston, TX, USA). Cells were cultured in P0 until day 10 with medium changes every other day. On day 10, cells were detached using TrypLE Express (Thermo Fisher Scientific Inc., Waltham, MA, USA) and passed through a cell strainer. To determine the percentage of differentiated cells, CD105 expression was measured using flow cytometry. Therefore, 5 × 10^4^ cells were resuspended in 50 µL FACS buffer (PBS + 0.1% BSA + 0.1% sodium azide) with 4% Gamunex (Grifols Deutschland GmbH, Frankfurt, Germany) and 5 µL of CD105-APC antibody (BioLegend, San Diego, CA, USA) was added. After 15 min incubation on ice, the samples were washed twice and then resuspended in 200 µL FACS buffer. CD105 expression was measured using a Guava EasyCyte 6HT-2L instrument (Merck Millipore, Billerica, MA, USA). The percentage of CD105^+^ cells was calculated and used to seed 1 × 10^6^ CD105^+^ cells in a T75 flask containing 10 mL hPL5. In the following passages, cells were maintained in hPL5 medium with medium changes every 2–3 days and passaged when reaching >80% confluency.

### 2.3. Flow Cytometric Analysis of MSC-Markers

The expression of MSC-markers (CD29, CD73, CD90, CD105) was analyzed by flow cytometry. Cells were detached using TrypLE Express and 1 × 10^5^ cells per sample were incubated on ice for 15 min in 20 µL blocking buffer (PBS, 0.1% BSA, 0.1 mg/mL sodium azide (Sigma-Aldrich, St. Louis, MO, USA) and 10% Gamunex (human immune globulin solution, Talecris Biotherapeutics GmbH, Frankfurt, Germany)). Then, 50 µL FACS buffer (PBS, 0.1% BSA, 0.1 mg/mL sodium azide) as well as phycoerythrin (PE) and allophycocyanin (APC)-conjugated antibodies (as listed in the Supplementary Table S1) were added and incubated on ice for 20 min. After two washing steps with 200 µL FACS buffer, flow cytometry measurements were performed using the Guava EasyCyte 6HT-2L instrument (Merck Millipore, Billerica, MA, USA).

### 2.4. Gene Expression Analyses

RNA isolation from JPCs and iMSCs was performed using the NucleoSpin RNA kit (Macherey-Nagel, Düren, Germany) following the manufacturer’s instructions. RNA concentration was measured. A total of 0.5 μg of RNA was used for first-strand cDNA synthesis using the SuperScript Vilo Kit (Thermo Fisher Scientific Inc., Waltham, MA, USA). The quantification of mRNA expression levels was performed using the real-time LightCycler System (Roche Diagnostics, Mannheim, Germany). For the PCR reactions, commercial primer kits (Search LC, Heidelberg, Germany), and DNA Master SYBR Green I (Roche, Basel, Switzerland) were used. The amplification was performed with a touchdown PCR protocol of 40 cycles (annealing temperature between 68 and 58 °C), following the manufacturer’s instructions. Copy numbers of each sample were calculated on the basis of a standard curve (standard included in the primer kits) and normalized to the housekeeping gene glyceraldehyde-3-phosphate dehydrogenase (GAPDH).

### 2.5. Osteogenic Differentiation

To stimulate osteogenic differentiation, iMSCs were cultivated in osteogenic medium (DMEM/F12 + 10% hPL (PL BioScience, Aachen, Germany), 1% Pen-Strep, 1% amphotericin B, 0.1 mM L-ascorbic acid 2-phosphate (Sigma-Aldrich, St. Louis, MO, USA), β-glycerophosphate (AppliChem, Darmstadt, Germany), and 4 µM dexamethasone (Sigma-Aldrich, St. Louis, MO, USA)) with medium changes every other day. Cultivation was performed for at least 15 days, but no longer than 25 days for Alizarin staining. After this cultivation time, cells were fixed with 4% formalin and monolayers were stained with Alizarin red solution (40 mM, pH 4.2) for 20 min. Unbound dye was washed off with distilled water and images were taken using an inverted microscope (Leica, Wetzlar, Germany). Quantification of calcium phosphate precipitates stained with Alizarin red was performed as previously reported [12]. Briefly, bound Alizarin dye was solubilized with 10% acetic acid and the absorbance at 405 nm was quantified photometrically after neutralization with 10% NH_4_OH.

### 2.6. Atomic Force Microscopy (AFM) Measurements

The stiffness of the cells and the resultant CaP precipitates were assessed using an AFM system (CellHesion200, Bruker, Billerica, MA, USA) combined with an inverted light microscope (Carl Zeiss Microscopy, Oberkochen, Germany). This setup enabled real-time visualization of the cells during measurements, as well as precise, region-specific AFM indentations. Microscale indentation was performed using a spherical cantilever with a 5 µm spherical tip (model: SAA-SPH-5UM, k = 0.2 N/m, Bruker). The cantilever was calibrated based on the extended curve, with the spring constant determined using the thermal noise method integrated into the device software (Bruker). Prior to AFM measurements, the cells were covered with Leibovitz’s L-15 medium without l-glutamine (Merck KGaA, Darmstadt, Germany), and the AFM microindentation measurements were performed at 37 °C. In force spectroscopy mode, force–distance curves were recorded at a sampling frequency of 2 kHz, employing a force trigger of 10 nN and a velocity of 5 µm/s. To evaluate the stiffness of JPCs and iMSCs, indentations were performed on selected cells identified through microscopic examination (three repetitions/cell; at least 30 cells per each cell type (JPCs/iMSCs)) and condition (untreated/osteogenic/hypoxia/normoxia/laminin/gelatin coating). The cantilever was positioned ideally in the middle of the cell. The 5 µm spherical tip of the cantilever transmits a cumulated Young’s modulus of cell cytoplasm and cell nucleus. Cell stiffness, expressed as Young’s modulus (elastic modulus = stress/strain), was computed from force–distance curves using the Hertz fit model for spherical indenters (indentation depth 0.5 µm, below 10% of the sample height) integrated within the data processing software (version 5.0.86, Bruker).

### 2.7. Fluorescent Staining of Osteogenically Differentiated JPC and iMSC Monolayers

Following AFM measurements, the cells were fixed using a fixation buffer (BioLegend) devoid of methanol to preserve actin filaments. Subsequently, the cells were permeabilized with PBS containing 0.1% Triton X-100 (AppliChem) for 5 min. Actin filaments were stained with phalloidin (1:100; green: PromoCell, Heidelberg, Germany; BioLegend; red: Thermo Fisher), while the nuclei were labeled using Hoechst (1:1000; PromoCell). For the detection of calcium phosphate (CaP) precipitates, calcein (1:1000; Sigma, USA) or xylenol orange (1:100; Carl Roth GmbH, Karlsruhe, Germany) was used. After incubation in the dark for 20–30 min, the cells were washed twice with PBS. Imaging was performed using an Axio Observer Z1 microscope (Carl Zeiss Microscopy) at 5×, 10×, and 20× magnifications, with phase contrast employed at 10× and 20× magnifications in Co dishes to enhance visualization.

### 2.8. Statistical Analysis

Histograms were employed to assess data normality. MSC surface marker expression, Alizarin quantification, and osteogenic marker gene expression were analyzed by calculating means ± standard deviations (SD) and compared using two-way ANOVA, followed by post hoc analysis with Tukey’s multiple comparison test. Statistical analyses were conducted using GraphPad Prism 10.1.1 software.

AFM results are presented as medians in boxplots, with group comparisons performed using the Kruskal–Wallis test, followed by the Mann–Whitney U test for post hoc analysis. The Kendall rank correlation coefficient was applied to evaluate the relationships between JPCs and iMSCs and various culture conditions, surface coatings, and biomechanical properties (Young’s modulus). Statistical analyses for the AFM data and correlation analysis were conducted using SPSS Statistics 22 (version 29.0.0.0, IBM Corp., Armonk, NY, USA). An alpha value of 0.05 was set as the threshold for statistical significance in all tests. JPC monolayers are very heterogeneous. Therefore, we averaged only the three repetitions of every AFM measurement of each cell. The periosteal tissue consists of a large proportion of tissue fibroblasts and a much lower proportion of mesenchymal progenitor cells. Due to the fact that the separation of tissue fibroblasts from the whole JPC population is not feasible, we treated all cells individually, performed independent measurements, and did not average data per donor but used all measured values from three donors for statistical significance calculations. iMSC populations should be more homogeneous than JPC populations but, for data comparability, we used the same calculation approach. Another aspect that has to be considered in this context is the asynchronous progress of differentiation in osteogenically induced cells, which has a great impact on data variability.

## 3. Results

### 3.1. Phenotypic Characterization of JPCs/iMSCs

For the characterization of the iMSC phenotype, MSC surface marker expression was analyzed by flow cytometry in comparison to that of their JPC predecessors (Supplementary Figure S1). Whereas CD29 and CD73 surface expression on iMSCs was almost 100%, as in JPCs, CD90 and CD105 surface expression showed higher variations between the three analyzed donors without reaching significant values.

Further, the mineralization potential of JPCs and iMSCs on different coatings was analyzed. Under normoxic conditions, JPCs and iMSCs were seeded onto 6-, 12-, and 24-well plates, and mineralization potential was investigated on uncoated plates versus laminin-521 and gelatin coatings (Supplementary Figure S2). In part A of the figure, it is obvious that the adhesion of iMSCs was impaired in uncoated plates. This problem was abolished in laminin- and gelatin-coated plates. In terms of mineralization, in the tendency, iMSC mineralization levels were shown to be lower than those detected for JPCs. Significant differences were recorded only for uncoated and gelatin-coated 12-well plates, as shown in part B of the figure.

Gene expression of osteogenesis-relevant genes (alkaline phosphatase and Runx-2) was detected in JPCs/iMSCs growing within uncoated plates (6- and 24-well) and laminin or gelatin coatings. As shown in Supplementary Figure S3, gene expression levels of ALPL and Runx-2 increased at day 10 (d10) compared to day 5 (d5), reaching only some condition levels of significance, as indicated in the figure. ALPL inducibility after 9/10 days (d9/d10) was much higher (note: algorithmic scale) in iMSCs compared to their JPC predecessors but the constitutive ALPL levels were, to some extent, lower in iMSCs compared to JPCs. Runx-2 expression levels were comparable in both cell types with the exception of the 24-well condition for JPCs.

### 3.2. Variability in Cellular Stiffness Between Undifferentiated JPCs and iMSCs

**Figure 2:**
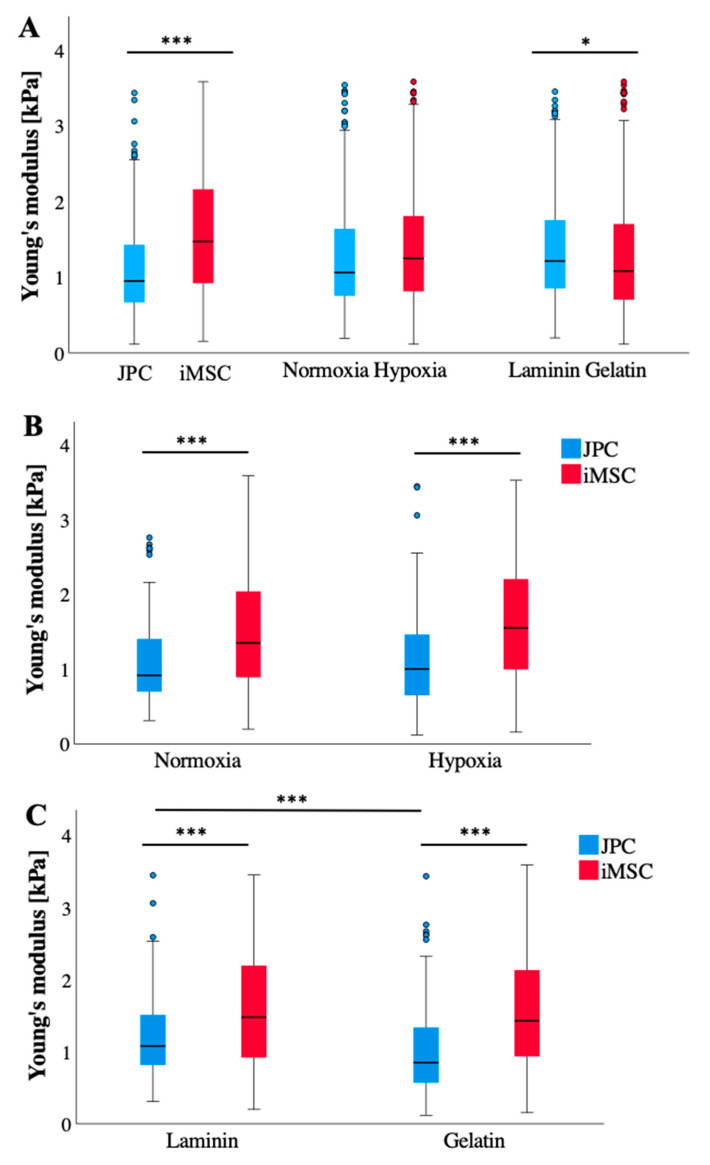
Young’s moduli of undifferentiated (Co) JPCs/iMSCs (n = 3) under different culture conditions, as measured by AFM measurements. (**A**) AFM data (n = 324–358 per boxplot) are itemized according to cell type (left), oxygen supply (normoxia/hypoxia, middle), and coatings (laminin, gelatin, right). (**B**) AFM data (n = 156–180) per boxplot) from (**A**) middle—normoxia/hypoxia boxplots—are broken down according to the cell type (JPCs/iMSCs). (**C**) AFM data (n = 160–180 per boxplot) from (**A**) right—laminin/gelatin boxplots—are broken down according to the cell type (JPCs/iMSCs). Data are shown as median (interquartile range) and T-shaped whiskers show maximal and minimal values. *p*-values of significance: * *p* < 0.05, *** *p* < 0.001.

Undifferentiated JPCs exhibited significantly lower cellular stiffness (*p* < 0.001) compared to iMSCs, and cells cultured on laminin showed significantly higher cellular stiffness (*p* = 0.016) compared to those growing on gelatin (Figure 2A). Across both cell types, hypoxic conditions tended to increase cellular Young’s moduli of iMSC relative to normoxic conditions (Figure 2B). Notably, the control (undifferentiated) JPCs displayed a significantly marked Young’s modulus reduction when cultured on gelatin-coated plates than on laminin substrates (Figure 2C).

### 3.3. Distinct Biomechanical Fingerprints of Untreated Cells, Osteogenically Induced Cells, and Formed CaP Precipitates

**Figure 3:**
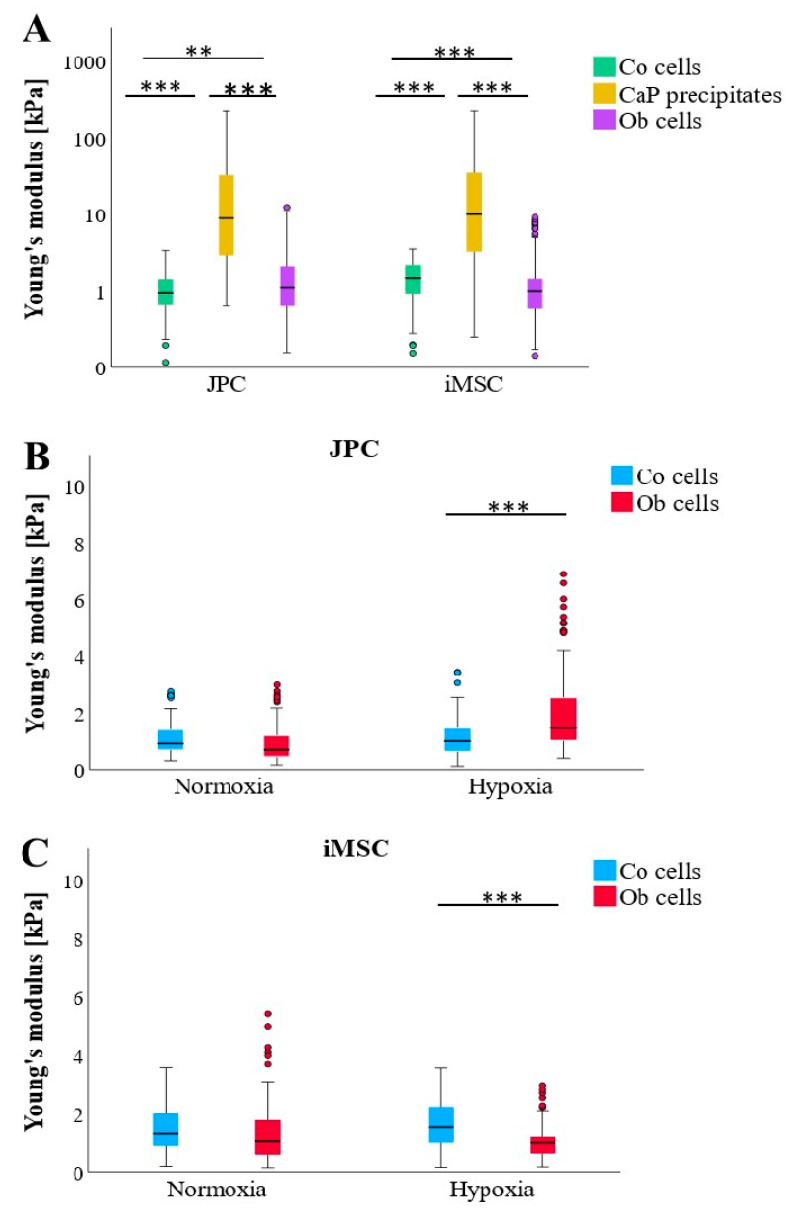
Comparison of Young’s moduli of undifferentiated (Co), osteogenically differentiated (OB) JPCs/iMSCs, and formed CaP precipitates (CaPP) under osteogenic conditions. (**A**) Comparison of cellular (Co and Ob) and CaPP stiffness measured in JPC and iMSC monolayers (n = 3) regardless of culture conditions (n = 324–358 per boxplot). (**B**) JPC (Co/Ob) stiffness cultured under normoxic and hypoxic conditions (n = 174–180 per boxplot). (**C**) iMSC (Co/Ob) stiffness cultured under normoxic and hypoxic conditions (n = 156–179 per boxplot). Data are shown as median (interquartile range) and T-shaped whiskers show maximal and minimal values. *p*-values of significance: ** *p* < 0.01, *** *p* < 0.001.

CaP depositions exhibit significantly higher stiffness compared to Co (untreated) and OB (osteogenically induced) cells (Figure 3A). Notably, while undifferentiated JPCs (also used as a control) are substantially softer than osteogenically induced JPCs, the opposite trend is observed in iMSCs, where undifferentiated cells display significantly greater stiffness profiles than osteogenically induced iMSC (ObiMSCs). This contrasting biomechanical behavior between JPCs and iMSCs becomes particularly pronounced under hypoxic conditions (Figure 3B,C). Furthermore, Young’s modulus for control iMSC (CoiMSC) is significantly higher than that of control JPC (CoJPC) (Figure 3A).

### 3.4. Influence of Culture Conditions and Substrate Coatings on Stiffness of the Mineralized Matrix Produced by JPCs and iMSCs

**Figure 4:**
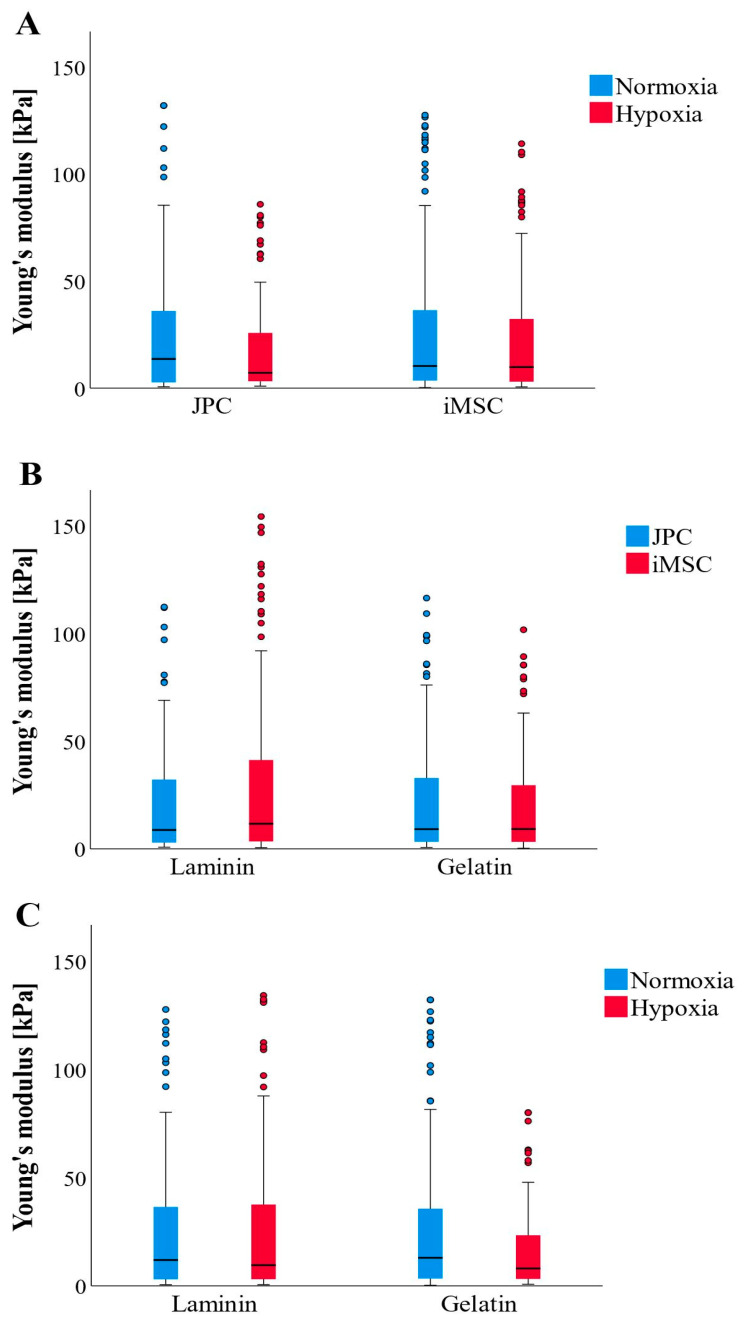
AFM-based Young’s moduli of formed CaP precipitates (CaPPs) (n = 177–179 per boxplot) after osteogenic differentiation of JPC and iMSCs (n = 3) under different culture conditions. (**A**) Comparison of CaPP stiffness formed by JPCs and iMSCs cultured under normoxic and hypoxic conditions. (**B**) Comparison of CaPP stiffness formed by JPCs and iMSCs cultured on laminin and gelatin-coated culture dishes. (**C**) Comparison of CaPP stiffness formed by both cell types (JPCs + iMSCs) cultured under different oxygen supply and coatings. Data are shown as median (interquartile range) and T-shaped whiskers show maximal and minimal values.

The measured Young’s moduli of the formed CaP precipitates did not show significant differences; however, observable trends were noted (Figure 4A–C). Overall, JPCs produced stiffer CaP precipitates (1.9-fold change) under normoxic conditions, while iMSCs exhibited greater Young’s moduli (1.3-fold change) in CaP depositions on laminin-coated plates. Overall, among all the tested condition combinations, JPCs cultured under normoxia produced the stiffest precipitates, with the coating showing no significant impact. In contrast, the softest precipitates were formed by JPCs under hypoxia. The precipitates from iMSCs exhibited an intermediate level of stiffness, falling between the two extremes (Figure 4A).

### 3.5. Influence of Culture Conditions and Substrate Coatings on Cellular Stiffness Profiles of Osteogenically Induced JPCs and iMSCs

**Figure 5:**
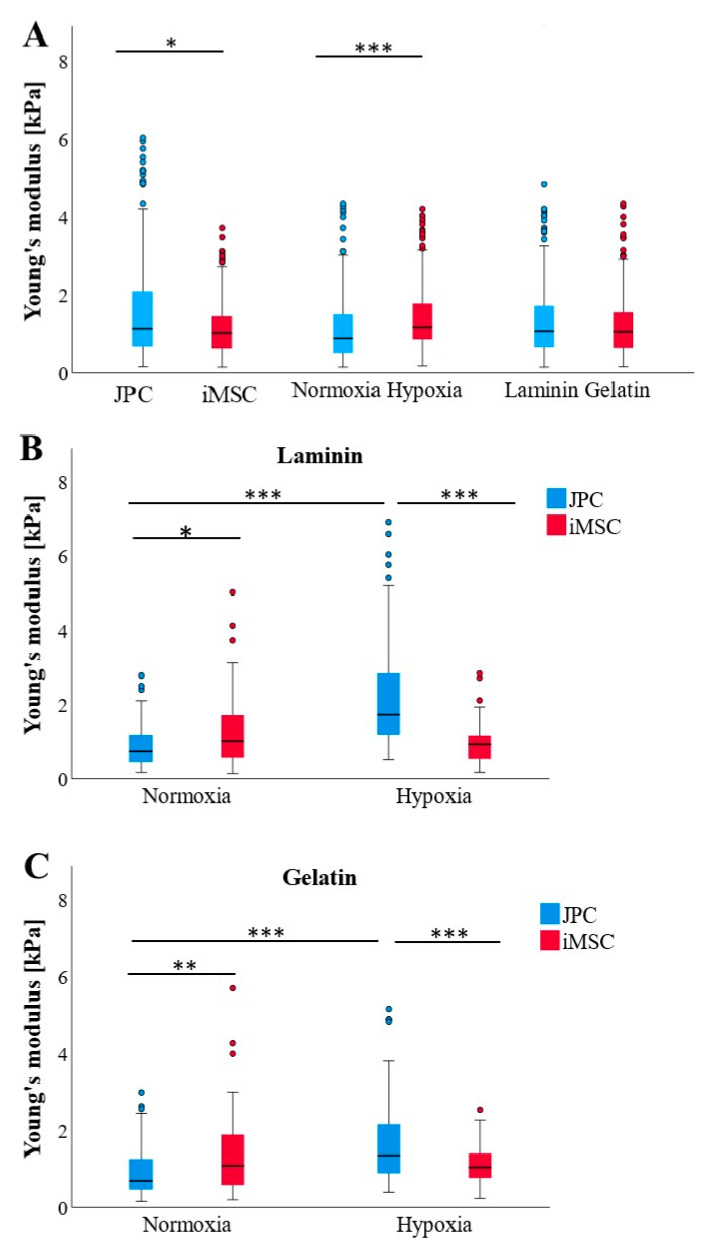
AFM-based Young’s moduli of osteogenically differentiated (Ob) JPCs/iMSCs (n = 3) under different culture conditions. (**A**) AFM data (n = 345–358 per boxplot) are itemized according to cell type (left), oxygen supply (normoxia/hypoxia, middle), and coatings (laminin, gelatin, right). (**B**) AFM data (n = 88–90 per boxplot) from laminin coatings were broken down according to the cell type (JPCs/iMSCs) and oxygen supply (normoxia/hypoxia). (**C**) AFM data (n = 83–90 per boxplot) from gelatin coatings were broken down according to cell type (JPCs/iMSCs) and oxygen supply. Data are shown as median (interquartile range) and T-shaped whiskers show maximal and minimal values. *p*-values of significance: * *p* < 0.05, ** *p* < 0.01, *** *p* < 0.001.

Osteogenically induced JPCs (ObJPC) exhibited significantly higher cellular stiffness profiles than differentiated iMSCs (Figure 5A). Hypoxic conditions appeared to markedly increase cell stiffness compared to normoxic conditions. Notably, JPCs and iMSCs cultured on laminin or gelatin-coated dishes did not show differences in Young’s moduli (Figure 5A). When accounting for cell type, ObJPCs had significantly lower Young’s moduli than ObiMSCs under normoxic conditions, while they displayed significantly greater stiffness than iMSCs under hypoxic conditions on both laminin and gelatin-coated plates (Figure 5B,C). Additionally, ObJPCs were significantly softer under normoxic compared to hypoxic conditions (Figure 5B,C). Considering the absolute values, ObJPCs under hypoxia showed the highest, and ObJPCs under normoxia showed the lowest Young’s moduli, while all Ob iMSCs were in between these two extremes.

### 3.6. Fluorescence Labelling of Cell Mineralization and Cytoskeleton Arrangement

**Figure 6:**
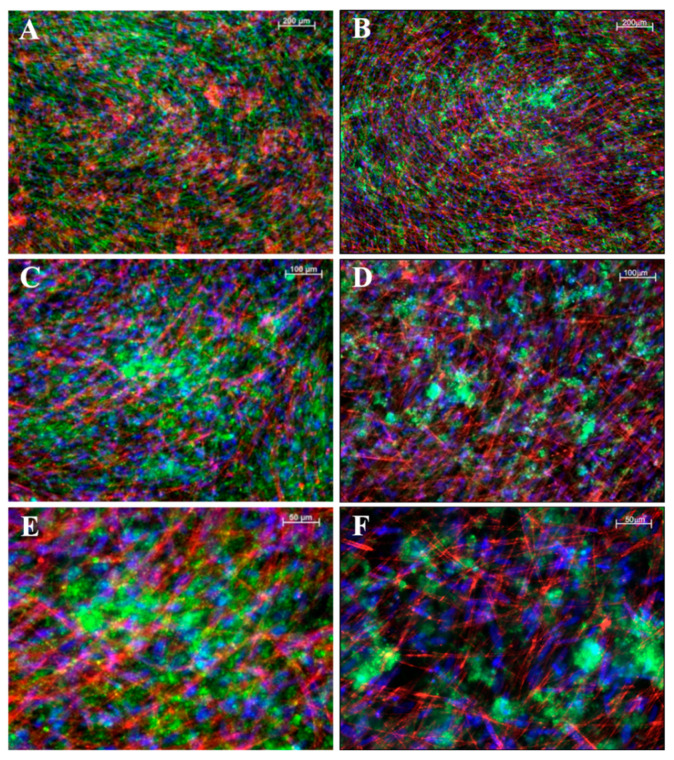
Fluorescent images of mineralized iMSC monolayers. (**A**,**B**) Images at 5× magnification, (**C**,**D**) at 10×, and (**E**,**F**) at 20× magnification. (**A**,**C**,**E**) iMSCs cultured on a gelatin substrate. (**B**,**D**,**F**) Cells cultured on laminin substrates. In panel (**A**), F-actin is shown in green and CaPP in red; in panels (**B**–**F**), F-actin is shown in red and CaPP in green. Cell nuclei stained with DAPI (blue). Scale bar depicted in white ((**A**,**B**): 200 µM; (**B**,**C**): 100 µM; (**E**,**F**): 50 µM).

**Figure 7:**
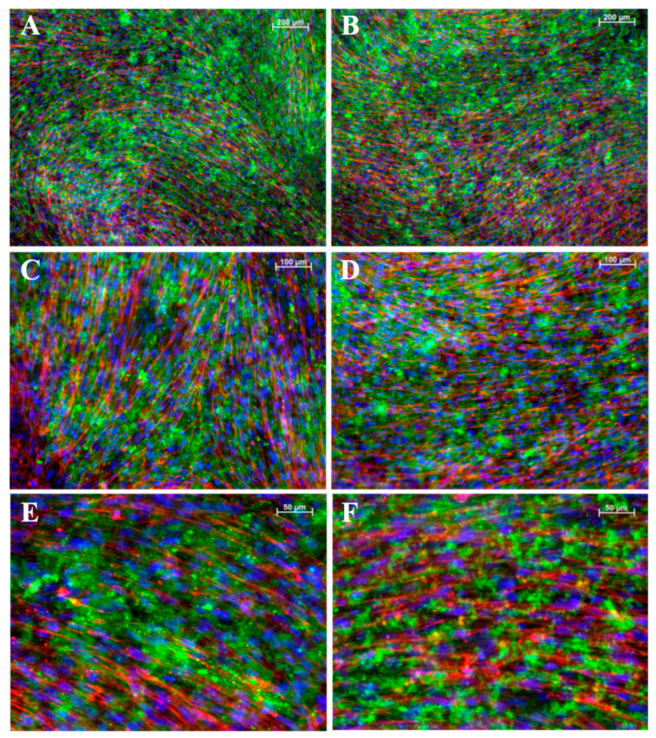
Fluorescent images of mineralized JPCs monolayers. (**A**,**B**) Images at 5× magnification, (**C**,**D**) at 10×, and (**E**,**F**) at 20× magnification. (**A**,**C**,**E**) iMSCs cultured on a gelatin substrate. (**B**,**D**,**F**) Cells cultured on laminin substrates. In all panels, F-actin is shown in red and CaPP in green. Cell nuclei stained with DAPI (blue). Scale bar depicted in white ((**A**,**B**): 200 µM; (**B**,**C**): 100 µM; (**E**,**F**): 50 µM).

To assess structural changes in iMSCs (Figure 6) and JPCs (Figure 7) under varying culture conditions, we employed fluorescent labeling to visualize CaP precipitates and the distribution of the F-actin cytoskeleton. This approach allowed us to directly observe the impact of the different conditions on cellular architecture and mineral deposition. While the CaPPs of JPCs exhibit an even distribution with fine granularity, the CaPPs of iMSCs are localized around distinct germinal centers, giving them a more pronounced, granular appearance.

**Figure 8:**
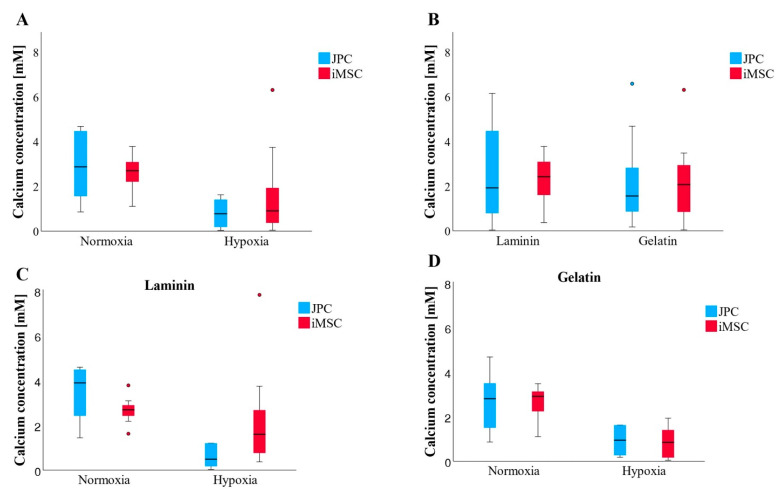
Quantification of cell mineralization by photometric measurements of the Alizarin staining. (**A**,**B**) Overview of all incubation conditions. (**A**) Both JPCs and iMSCs generated larger quantities of CaPPs under normoxic conditions, with results across coatings averaged in (**B**) (n = 12–14 per boxplot). (**C**) Under laminin and normoxia, JPCs produced more CaPPs (n = 6–7 per boxplot) than iMSCs, whereas, under laminin and hypoxia, iMSCs outperformed JPCs in CaPP production. (**D**) On gelatin, both in normoxic and hypoxic conditions, iMSCs and JPCs produced comparable amounts of CaPPs (n = 6–7 per boxplot). Data are shown as median (interquartile range) and T-shaped whiskers show maximal and minimal values.

The measured quantities of CaP precipitates (CaPP) did not exhibit significant differences; however, discernible trends were observed (Figure 8A,B). Regardless of the coating or cell type, cells produced the highest amounts of CaPP under normoxic conditions (Figure 8C,D). Additionally, iMSCs appeared to synthesize more CaPP than JPCs on laminin or gelatin substrates. Interestingly, under normoxic conditions on laminin, JPCs exhibited higher numbers of CaPP compared to iMSCs.

### 3.7. JPC and iMSC Mineralization Potential Correlates with Cellular Biomechanical Fingerprint

A negative correlation was observed between the stiffness of CaP precipitates and Young’s moduli of ObJPCs, although this did not reach statistical significance (Table 1). In contrast, a very weak positive correlation was noted between the stiffness of CaP precipitates and the stiffness of ObiMSCs, again lacking significance. Notably, a significant negative correlation emerged between the stiffness of Ob cells in the combined group JPCs + iMSCs and the extent of cell mineralization (row calcium concentration vs. Young’s modulus, right panel). Additionally, a highly significant correlation was found between the stiffness of ObJPCs and the degree of cell mineralization (row calcium concentration vs. Young’s modulus, left panel).

## 4. Discussion

To overcome the replicative limitations and age-related decline of jaw periosteal cells (JPCs), we generated induced mesenchymal stem cells (iMSCs). However, a key question remains: do these iMSCs exhibit osteogenic potential comparable to, or better than, their JPC counterparts? Our study delves into the biomechanical properties of these stem cells and the bone matrix they produce under a range of culture conditions. By systematically comparing JPCs and iMSCs across different oxygen environments (normoxia and hypoxia) and substrate coatings (laminin and gelatin), we aimed to investigate how these factors shape cellular biomechanical fingerprints and matrix formation.

Young’s modulus of undifferentiated JPCs and iMSCs in our study was ~1 kPa, aligning with previous reports where values for MSCs ranged between 0.8 and 9 kPa [13,20,21]. In contrast, osteogenically differentiated cells typically exhibit moduli between 1.5 and 50 kPa, partially depending on the substrate [23,24]. These differences are likely attributable to variations in cell types, experimental conditions, and analytical techniques employed [22]. In our study, the substrate coating had a limited effect on Young’s modulus of the undifferentiated cells, with the notable exception that JPCs exhibited greater stiffness on laminin-coated plates (Figure 2C) compared to those coated with gelatin. Besser and colleagues demonstrated that cells form more stable bonds with laminin than with pure gelatin substrates [25].

Interestingly, during osteogenic differentiation, iMSCs exhibit a distinct biomechanical fingerprint characterized by a lower Young’s modulus compared to JPCs. The difference in Young’s modulus between untreated Co and osteogenically differentiated Ob cells is statistically significant for both JPCs and iMSCs. While Yen et al. demonstrated that MSCs increase in stiffness during osteogenic differentiation [16], Titushkin et al. reported an opposing finding, showing that human MSCs possess a stiffer profile of 3.2 ± 1.4 kPa compared to fully differentiated osteoblasts, which measure only 1.7 ± 1.0 kPa [22]. Their results also indicated a reduction in Young’s modulus to 2.1 ± 0.9 kPa after 10 days of osteogenic induction in human MSCs, representing a 34.4% decrease from baseline measurements [22]. These findings align with the results of this present study for iMSCs, which demonstrated a median Young’s modulus of 1.5 ± 0.8 kPa, decreasing to a median of 1.0 ± 1.4 kPa after 15 days of osteogenic differentiation, reflecting a 33.3% reduction compared to the pre-differentiation value. Out of all observed groups, JPCs cultured under hypoxia were the only cells that gained stiffness during the process of osteogenic differentiation. In this context, Ob cells under hypoxic conditions exhibited significantly greater stiffness compared to those cultured under normoxic conditions. This phenomenon may be attributed to oxygen stress, which promotes the formation of additional actin stress fibers in hypoxic environments [26].

As already mentioned in the introduction, the highest oxygen tension was detected within the periosteum due to its high vascularization degree, and therefore, we presume that JPCs are quite susceptible to low oxygen conditions. Our experience shows that JPCs increase in senescence under hypoxic conditions. The significant increase in the stiffness of JPCs detected in our present study could be based on both oxygen stress and the occurrence of cell senescence since a recent study of Brauer and colleagues showed evidence that senescent cells exhibited increased cell stiffness and even caused the contraction of 3D collagen scaffolds colonized with p16 and p21-overexpressing cells [27]. In our study, higher values for both the number and stiffness of CaP precipitates were observed in favor of normoxia. Only iMSCs cultured on laminin demonstrated no difference between normoxia and hypoxia. In addition to that, iMSCs seemed to manage the lack of oxygen better than their parental JPCs pre-primed with a high oxygen supply of the periosteum tissue.

Concerning the influence of coating on cell differentiation outcomes, no significant differences were observed between laminin and gelatin coatings regarding Young’s modulus or the quantity of CaP precipitates formed. Both coatings demonstrated comparable effectiveness in terms of the resulting quantity and stiffness of the CaP precipitates. Notably, gelatin appeared slightly more favorable for JPCs under hypoxic conditions, while iMSCs in the same conditions showed a trend toward producing firmer and larger amounts of CaP precipitates on laminin.

Alizarin quantification demonstrates significant variability in mineralization levels among individual patients, as well as notable differences between iMSCs and JPCs derived from the same patient. Although the data did not achieve statistical significance, they suggest trends that align with Young’s modulus of the CaP precipitates, indicating that iMSCs typically exhibit superior mineralization compared to JPCs. This trend was consistent under both hypoxic conditions and across laminin and gelatin substrates. Interestingly, JPCs outperformed iMSCs in mineralization solely under normoxic conditions. On laminin-coated plates, iMSCs successfully produced hard precipitates in both normoxic and hypoxic environments, highlighting their robust mineralization capacity and adaptability across varying conditions.

A significant correlation between the elastic modulus of Ob cells (overall and also for JPCs without iMSCs) and the amount of cell mineralization was calculated. This relationship was negatively proportional, meaning that the softer the Ob cells, the more CaP precipitates they formed. This again supports the aforementioned theory that fully differentiated Ob cells are more elastic than their undifferentiated predecessors, and the better the cells are differentiated, the more mineralized ECM they can form.

Titushkin and Cho attribute the inherited changes in biomechanical properties to the interaction between the cell membrane and the F-actin cytoskeleton [22]. Gavara and Chadwick found a strong correlation between actin and myosin fiber amount and cellular elasticity, whereby the slope obtained for myosin was significantly larger than the value obtained for actin. This result is based on the fact that actin comprises the bulk of the filamentous scaffold of the cell, whereas myosin minifilaments reinforce and add tension to the structure [28]. LeBlon and colleagues further demonstrate that the thickness of actin fibers correlates with Young’s modulus of the cell [29]. They propose that osteoblasts are regularly subjected to various mechanical stresses, which they manage through elastic deformability. Although both cell types examined in our work appear to have a similar quantity of F-actin fibers, these fibers are arranged into a significantly finer network in Ob cells, explaining the lower Young’s moduli of Ob compared to Co cells. Given that the JPCs originate from the mechanically stressed mandible, they are likely subjected to high mechanical forces [30] and may have adapted to these conditions by modulating their inherited Young’s moduli. Conversely, undifferentiated JPCs (CoJPCs) exhibited an increase in fat vacuole formation over time with 10% hPL supplementation, a phenomenon not observed in iMSCs. This difference may also contribute to the observed softness of CoJPCs compared to CoiMSCs.

The AFM measurements revealed that Young’s moduli of JPCs, in relation to CaP precipitates, reach either peak values under normoxia or drop to minima under hypoxia. In contrast, iMSCs consistently produce moderate precipitate hardness across all conditions. Since hypoxic conditions tend to prevail in real clinical applications [31] and an oxygen content < 1% to 6% is considered physiological in vital bone [32], iMSCs outperform JPCs, exhibiting higher Young’s moduli and generating larger quantities of CaP precipitates, as mentioned before. Further, we provided evidence that the softer the osteogenically differentiated cells, the more mineralized matrix they form.

Overall, the AFM approach stands out as an exceptional method for assessing cellular elasticity. To tackle potential challenges, particularly with fully mineralized cell monolayers, future studies should focus on increasing measurement frequency and leveraging full automation to minimize time-related inconsistencies.

## 5. Conclusions

Among the tested culture conditions, both oxygen supply and differentiation state rather than coating conditions provoke different cellular elasticities, indicating different biomechanical fingerprints in iMSCs versus JPCs. Further, we provided evidence that a lower cellular stiffness profile in both cell types correlates with a higher degree of mineralization, indicating that cellular stiffness represents a critical marker for osteogenic differentiation.

Further studies should focus on the underlying mechanisms in order to understand the fundamental differences between iMSCs and their predecessors and to sharpen their use in future clinical applications, possibly considering the defect localization, such as compact or trabecular bone.

## Figures and Tables

**Table 1:** A—Correlation analysis of Young’s moduli (CaPP vs. Ob, CaPP vs. Co, and Co vs. Ob) of JPCs, iMSCs separately, or in combination (JPCs + iMSCs) across different culture dish coatings. B—Correlation analysis of calcium concentration and Young’s moduli across different culture dish coatings and oxygen supply. Abbreviations: Co—control, undifferentiated cells, Ob—osteogenically induced, JPC—jaw periosteal cells, iMSC—induced mesenchymal stem cells, t—Kendall’s Tau coefficient.

A						
Cell Type	JPCs		iMSCs		JPCs + iMSCs	
**Young’s modulus**	**CaPP vs. Ob** t = −0.121, *p* = 0.583 **CaPP vs. Co** t = 0.091, *p* = 0.681 **Co vs. Ob** t = 0.364, *p* = 0.100		**CaPP vs. Ob** t = 0.091, *p* = 0.681 **CaPP vs. Co** t = −0.212, *p* = 0.337 **Co vs. Ob** t = 0.333, *p* = 0.131		**CaPP vs. Ob** t = 0.043, *p* = 0.766 **CaPP vs. Co** t = −0.094, *p* = 0.519 **Co vs. Ob** t = 0.225, *p* = 0.124	
**Coating** **1. CaPP vs. Ob**	**Laminin** t = −0.333 *p* = 0.348	**Gelatin** t = 0.200 *p* = 0.573	**Laminin** t = −0.033 *p* = 0.348	**Gelatin** t = 0.333 *p* = 0.348	**Laminin** t = −0.152 *p* = 0.493	**Gelatin** t = 0.303 *p* = 0.170
**2. CaPP vs. Co**	t = 0.200 *p* = 0.573	t = 0.067 *p* = 0.851	t = −0.467 *p* = 0.188	t = −0.200 *p* = 0.573	t = −0.152 *p* = 0.493	t = 0.030 *p* = 0.891
**3. Co vs. Ob**	t = 0.467 *p* = 0.188	t = 0.333 *p* =0.348	t = 0.333 *p* = 0.348	t = 0.200 *p* = 0.573	t = 0.152 *p* = 0.493	t = 0.303 *p* = 0.891
**B**						
**Cell Type**	**JPCs**		**iMSCs**		**JPCs + iMSCs**	
**Calcium concentration (cc) vs. Young’s modulus (Ym)**	**CaPP** t = 0.242, *p* = 0.273 **Ob** t = −0.636, *p* = 0.004 **Co** t = −0.152, *p* = 0.49		**CaPP** t = 0.273, *p* = 0.217 **Ob** t = 0.091, *p* = 0.681 **Co** t = −0.212, *p* = 0.337		**CaPP** t = 0.232, *p* = 0.112 **Ob** t = −0.362, *p* = 0.013 **Co** t = −0.101, *p* = 0.487	
**Coating** **1. CaPP ** **Cc vs. Ym**	**Laminin** t = 0.200 *p* = 0.573	**Gelatin** t = 0.067 *p* = 0.851	**Laminin** t = 0.333 *p* = 0.348	**Gelatin** t = 0.333 *p* = 0.348	**Laminin** t = 0.242 *p* = 0.273	**Gelatin** t = 0.212 p= 0.337
**2. Ob** **Cc vs. Ym**	t = −0.867 *p* = 0.015	t = −0.600 *p* =0.091	t = 0.067 *p* = 0.851	t = 0.200 *p* = 0.573	t = −0.485 *p* = 0.028	t = −0.212 *p* = 0.337
**3. Co** **Cc vs. Ym**	t = −0.333 *p* = 0.348	t = −0.200 *p* = 0.573	t = −0.333 *p* = 0.348	t = −0.067 *p* = 0.851	t = −0.242 *p* = 0.273	t = 0.000 *p* = 1.000
**Culturing Condition** **1. CaPP** **Cc vs. Ym**	**Normoxia** t = −0.067 *p* = 0.851	**Hypoxia** t = 0.200 *p* = 0.573	**Normoxia** t = 0.200 *p* = 0.573	**Hypoxia** t = 0.200 *p* = 0.573	**Normoxia** t = 0.061 *p* =0.784	**Hypoxia** t = 0.121 *p* = 0.583
**2. Ob** **Cc vs. Ym**	t = −0.333 *p* = 0.348	t = −0.333 *p* = 0.348	t = −0.200 *p* = 0.573	t = 0.200 *p* = 0.573	t = −0.333 *p* = 0.131	t = −0.364 *p* = 0.100
**3. Co** **Cc vs. Ym**	t = −0.200 *p* = 0.573	t= −0.200 *p* = 0.573	t = −0.733 *p* = 0.039	t = 0.333 *p* = 0.348	t = −0.485 *p* = 0.028	t = 0.333 *p* = 0.131

## Data Availability

The data that support the findings of this study are available from the corresponding author upon reasonable request.

## Supplementary Materials

The following supporting information can be downloaded at: https://www.mdpi.com/article/10.3390/bioengineering11121282/s1, Figure S1: Surface marker expression of undifferentiated and differentiating JPCs and iMSCs on different coatings.; Figure S2: Mineralization of JPCs and iMSCs on different coatings; Figure S3: Quantitative gene expression of ALPL and RUNX2 in JPCs and iMSCs; Table S1: List of antibodies (antigen, isotype, conjugate and producer) used for flow cytometry.
