# Supplementary material for: Mesenchymal Stem Cell Plasticity: What Role Do Culture Conditions and Substrates Play in Shaping Biomechanical Signatures?
Source: Bioengineering (Basel). 2024 Dec 17;11(12):1282. doi: 10.3390/bioengineering11121282 (PMC11673249; doi:10.3390/bioengineering11121282)
Supplement: Supplementary file 1 [file bioengineering-11-01282-s001.zip › bioengineering-3345355-supplementary.pdf]

1 Suplementary material

MSC marker surface expression of JPCs/iMSCs cultured on uncoated, gelatin- and laminin-coated plates

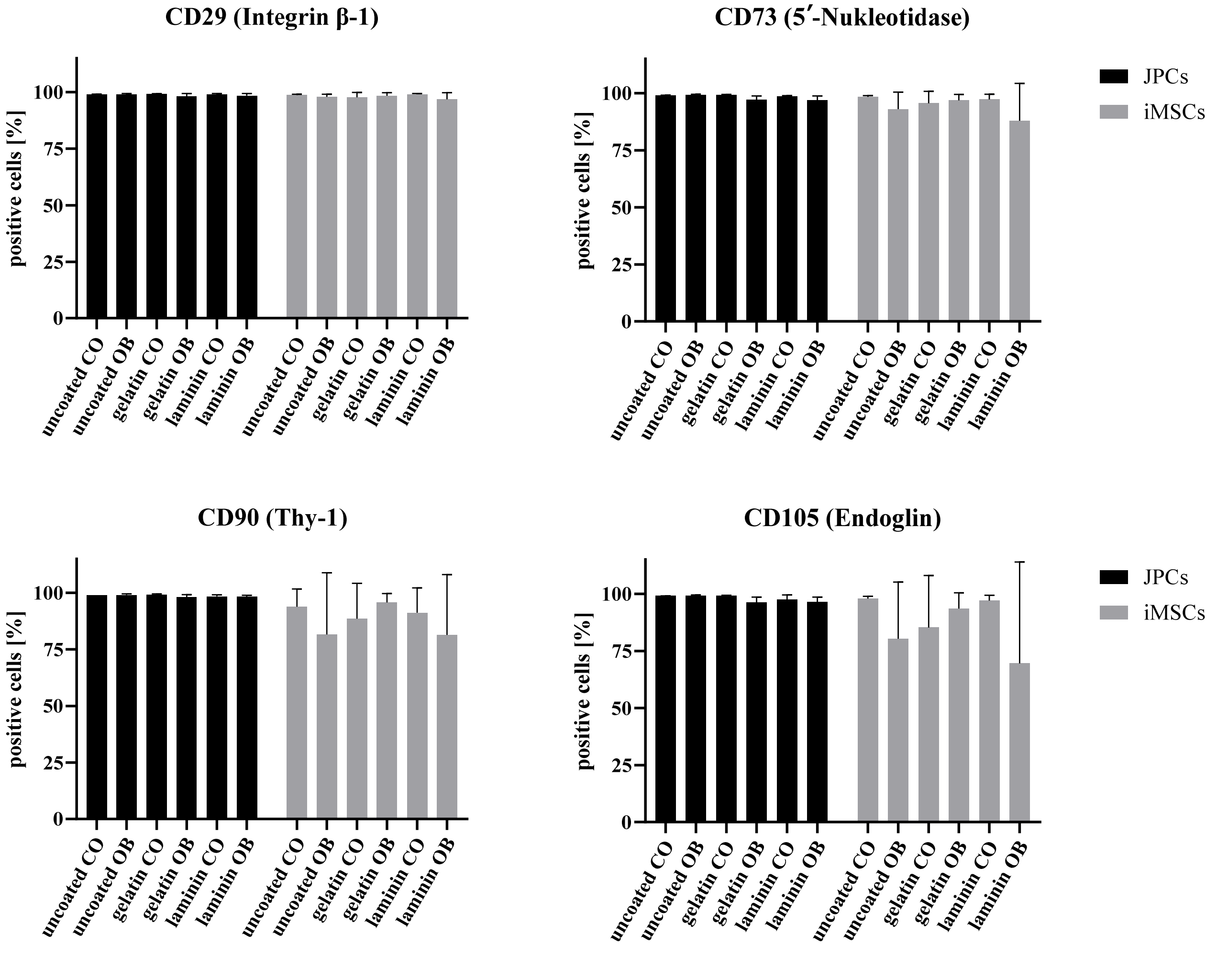

Figure S1: Surface marker expression of undifferentiated and differentiating JPCs and iMSCs on different coatings. The expression of the surface markers CD29, CD73, CD90, and CD105 was assessed after five days of cultivation under normal (CO) and osteogenic (OB) conditions on different coatings in 6-well plates. Surface marker expression was measured by flow cytometry and is displayed as percentage of positive cells minus isotype control. Data are presented as mean ± standard deviation and compared using two-way ANOVA and Tukey's multiple comparison test (n = 3).

Table 1: List of antibodies (antigen, isotype, conjugate and producer) used for flow cytometry

<table>
<tr><th>Human Antigen</th><th>Isotype</th><th>Conjugate</th><th>Company</th></tr>
<tr><td>CD29</td><td rowspan="6">mouse IgG1</td><td>PE</td><td rowspan="6">BioLegend, San Diego, USA</td></tr>
<tr><td>CD73</td><td>APC</td></tr>
<tr><td>CD90</td><td>APC</td></tr>
<tr><td>CD105</td><td>APC</td></tr>
<tr><td>IgG1 - Isotype</td><td>PE</td></tr>
<tr><td>IgG1- Isotype</td><td>APC</td></tr>
</table>

Mineralization of JPCs/iMSCs cultured on uncoated, gelatin-, and laminin-coated plates

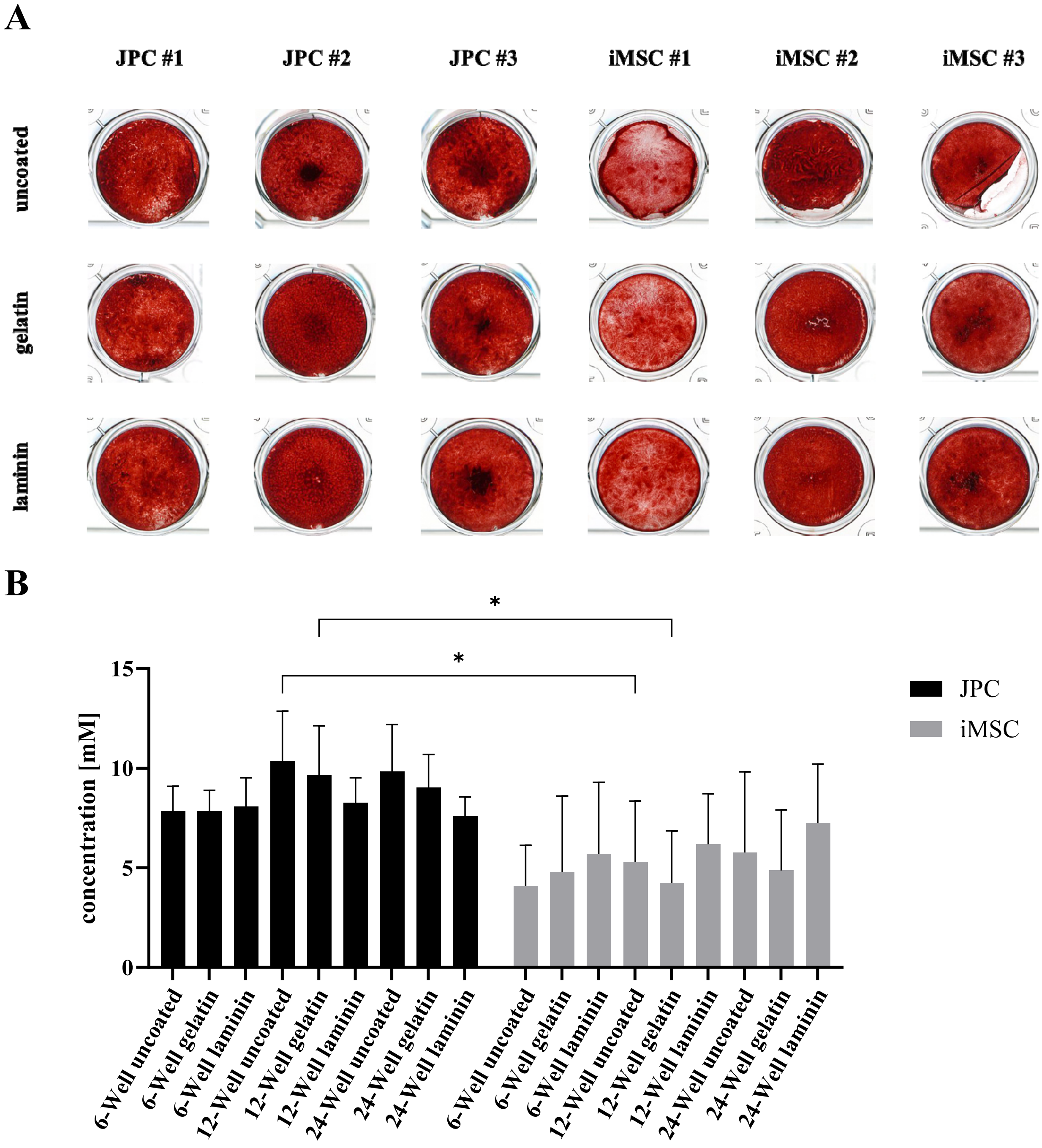

Figure S2: Mineralization of JPCs and iMSCs on different coatings. A) Mineralization was analyzed following a 14-day period of osteogenic differentiation on three different plate formats. The illustration depicts representative photographic images of individual wells of osteogenically differentiated JPCs and iMSCs on uncoated wells (upper panel), 0.1% gelatin (middle panel) and laminin-521 (lower panel). Mineralization was visualized by alizarin red staining. B) Quantification of alizarin red staining. The mineralization of the differentiated JPCs and iMSCs was quantified photometrically and 6-, 12-, and 24-well plates as well as three different coatings were compared. The data are presented as the mean ± standard deviation. The groups were compared using two-way ANOVA and Tukey's multiple comparison test ($n \geq 2$, $*p < 0.05$).

Expression of osteogenic marker genes in JPCs/iMSCs cultured on uncoated, gelatin-, and laminin-coated plates

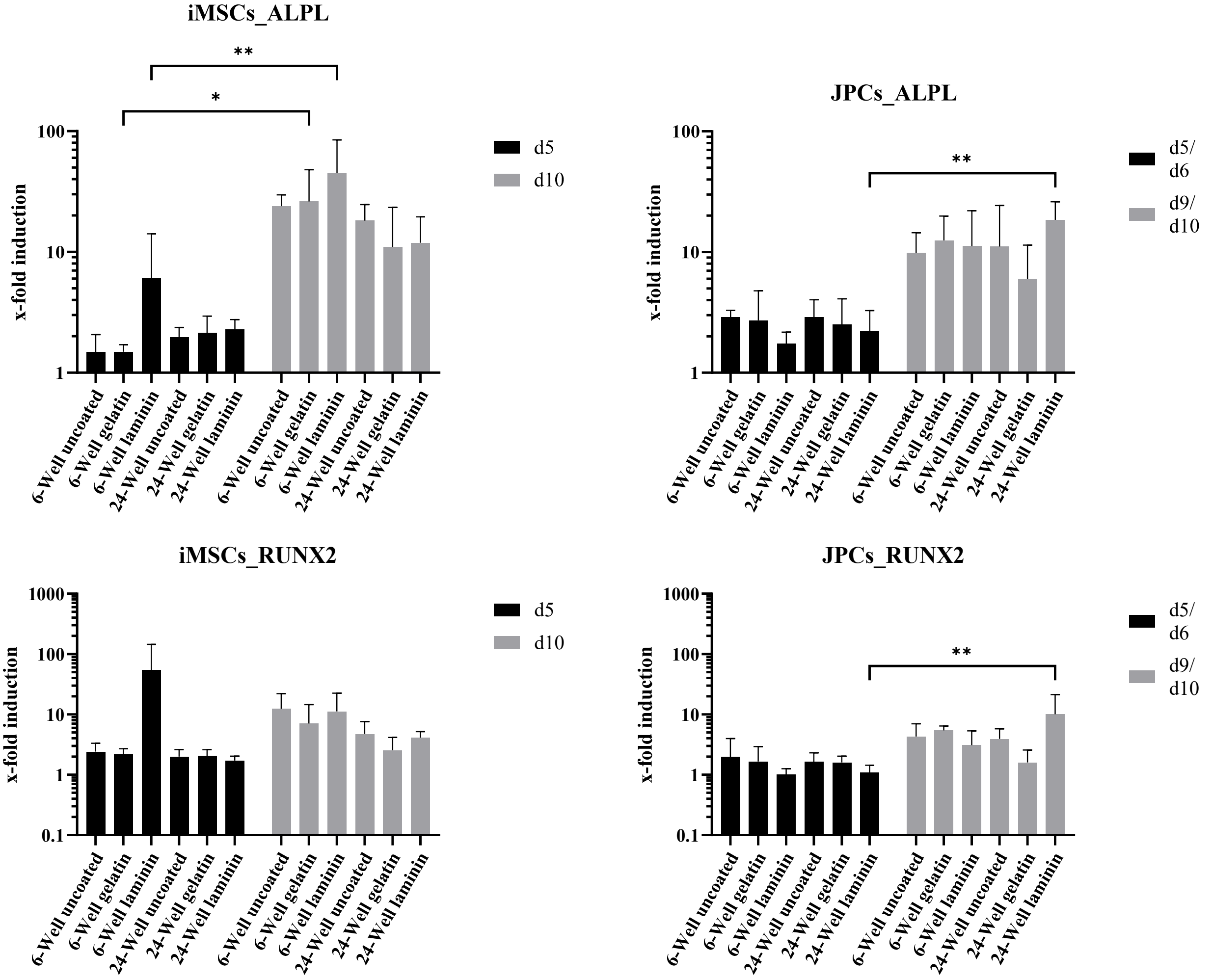

*Figure S3: Quantitative gene expression of ALPL and RUNX2 in JPCs and iMSCs. The expression of ALPL and RUNX2 in JPCs and iMSCs was analyzed under normoxic conditions following five- or six-day (d5, d6) and nine- or ten-day (d9, d10) cultivation periods under normal (CO) and osteogenic (OB) conditions on various coatings and on 6- and 24-well plates. The gene expression levels were normalized to the housekeeping gene GAPDH and are presented as x-fold inductions (logarithmic scale), relative to the corresponding undifferentiated control sample (CO). The data are presented as means ± standard deviation and groups were compared using two-way ANOVA and Tukey's multiple comparison test (n=3, *p < 0.05, **p ≤ 0.01).*
